# Obituary

**Published:** 1909-12-11

**Authors:** 


### Obituary.

The death is announced of Mr. Charles Robert Bell Keetley at Brighton at the age of 61, surgeon to the West London Hospital, of 56 Grosvenor Street, London, W. Mr. Keetley studied at St. Bartholomew's Hospital, and took his diploma in 1873. He won the gold medal in anatomy at London University, and in 1876 became a Fellow of the Royal College of Surgeons. He held the post of senior surgeon to the West London Hospital, and was consulting surgeon to the Brentford Hospital, the Cray Valley Cottage Hospital, and the Fleet Cottage Hospital. At one time he was President of the West London Medical and Chirurgical Society, and was the author of "An Index of Surgery," "The Students' Guide to the Medical Profession," "Orthopædic Surgery," and "Complicated Fractures," besides being formerly co-editor of "The Annals of Surgery."

The death is announced of Major Espine Charles Robert Ward, at the age of sixty-one. Major Ward, who studied at Coombe Hospital, Dublin, took his diploma in Ireland in 1870, becoming in 1883 Fellow of the R.C.S. of Ireland. He also joined the Army Medical Staff, from which he retired to his home at Castle Connel, Ireland.

